# Computed tomography of the equine temporohyoid joint: Association between imaging changes and potential risk factors

**DOI:** 10.1111/evj.14495

**Published:** 2025-05-05

**Authors:** Rupert F. Dash, Justin D. Perkins, Yu‐Mei Chang, Rhiannon E. Morgan

**Affiliations:** ^1^ The Royal Veterinary College, Equine Referral Hospital North Mymms Hertfordshire UK; ^2^ IVC Evidensia UK, The Chocolate Factory Bristol UK

**Keywords:** CT, diagnostics, horse, imaging, THO

## Abstract

**Background:**

Temporohyoid osteoarthropathy (THO) is characterised by bone proliferation and cartilage ossification caused by infectious and degenerative conditions, amongst others.

**Objectives:**

To describe the variable appearance of the temporohyoid joint (THJ) on computed tomography (CT) and investigate associations between CT changes and potential risk factors.

**Study Design:**

Cross‐sectional study.

**Methods:**

Head CT examinations were assessed. A grading system was developed for osseous proliferation (grade 0 [normal] to 3 [severe]) and tympanohyoid cartilage change (grade 0 [normal] to 3 [complete ossification]). Grades were also summed to create an overall sum grade. Ordinal logistic regression was performed to produce a multivariable model that assessed the association between THJ grade and signalment, presenting signs, CT features, and final diagnosis.

**Results:**

The horses included (*n* = 424) most commonly presented for dental and sinus disorders (37.7%). The most frequently observed (mode) bone grade, cartilage grade and overall grade were 2 (41.9%), 0 (52.6%) and 2 (27.0%), respectively. Bone proliferation was most common medially and caudally. Soft tissue swelling (OR 1.9, 95% CI 1.2–3.1, *p* < 0.05) and temporal bone fragmentation (OR 26.6, 95% CI 5.1–141.4, *p* < 0.05) were associated with increased bone grade. There was no correlation between increased grade and any presenting sign. Increased sum grade was significantly associated with increased age (OR per year 1.1, 95% CI 1.0–1.1, *p* < 0.05), Arabians (OR 4.2, 95% CI 1.3–14.0, *p* < 0.05) and Thoroughbreds (OR 2.9, 95% CI 1.5–5.4, *p* < 0.05) relative to Warmbloods.

**Main Limitations:**

Following training, a single observer evaluated images.

**Conclusions:**

Moderate caudomedial osseous proliferation of the THJ is common in horses presented for unrelated disease. Cartilage mineralisation, soft tissue swelling, and temporal bone fragmentation may serve as markers of disease. Thoroughbreds and Arabians are at increased risk of greater THJ remodelling. Increased THJ change was associated with age but not otitis, suggesting THO is predominantly degenerative.

## Zusammenfassung

**Hintergrund:**

Die Temporohyoid‐Osteoarthropatie (THO) ist durch Knochenproliferation und Verknöcherung des Knorpels gekennzeichnet, welche unter anderem durch infektiöse und degenerative Erkrankungen verursacht werden.

**Zielsetzung:**

Beschreibung des variablen Erscheinungsbildes des Temporohyoidgelenks (THJ) in der Computertomographie (CT) und Untersuchung von Zusammenhängen zwischen CT‐Veränderungen und möglichen Risikofaktoren.

**Studiendesign:**

Querschnittsstudie.

**Methodik:**

CT‐Untersuchungen des Kopfes wurden ausgewertet. Es wurde ein Bewertungssystem für die knöcherne Proliferation (Grad 0 [normal] bis 3 [schwerwiegend]) und die tympanohyoidale Knorpelveränderung (Grad 0 [normal] bis 3 [vollständige Verknöcherung]) entwickelt. Die Grade wurden auβerdem addiert, um einen Gesamtsummengrad zu erhalten. Es wurde eine logistische Ordinalregression durchgeführt, um ein multivariables Modell zu erstellen, das den Zusammenhang zwischen Grad des THJ und des Signalements, Vorstellungsgrund, CT‐Merkmalen und der endgültigen Diagnose beurteilte.

**Ergebnisse:**

Die einbezogenen Pferde (*n* = 424) wurden am häufigsten wegen Zahn‐ und Kieferhöhlenerkankungen vorgestellt (37,7%). Der am häufigsten beobachtete Knochengrad, Knorpelgrad und Gesamtsummengrad waren 2 (41,9%), 0 (52,6%) und 2 (27,0%). Knochenproliferationen traten am häufigsten medial und kaudal auf. Weichteilschwellungen (OR 1,9, 95% CI 1,2‐3,1, *p* < 0,05) und Knochenfragmentierung des Os Temporale (OR 26,6, 95% CI 5,1‐141,4, *p* < 0,05) waren mit einem erhöhten Knochengrad verbunden. Es gab keine Korrelation zwischen erhöhtem Grad und einem der Vorstellungsgründe. Ein erhöhter Summengrad war signifikant mit einem höherem Alter (OR pro Jahr 1,1, 95% CI 1,0‐1,1, *p* < 0,05), Arabern (OR 4,2, 95% CI 1,3‐14,0, *p* < 0,05) und Vollblütern (OR 2,9, 95% CI 1,5‐5,4, *p* < 0,05) im Vergleich zu Warmblütern verbunden.

**Haupteinschränkungen:**

Nach dem Training wurde ein einzelner Beobachter eingesetzt.

**Schlussfolgerung:**

Eine mäβige kaudomediale knöcherne Proliferation des THJ ist bei Pferden, die wegen einer nicht im Zusammenhang stehenden Krankheit vorstellig werden, häufig. Knorpelmineralisierung, Weichteilschwellung und Fragmentierung des Os Temporale können als Marker für Erkrankung dienen. Vollblüter und Araber haben ein erhöhtes Risiko für eine stärkere Umbildung des THJ. Vermehrte Veränderungen des THJ wurden mit dem Alter, nicht aber mit einer Otitis in Verbindung gebracht, was darauf hindeutet, dass die THO überwiegend degenerativ ist.

## 1 INTRODUCTION

The paired temporohyoid joints (THJ) are formed by the articulation of the dorsal aspect of the stylohyoid bone and the styloid process of the petrous temporal bone.^1^ These bones are separated by the small tympanohyoid cartilage, which is composed of fibrocartilage, making this joint a symphysis or secondary cartilaginous joint.^2^, ^3^ This joint acts as a hinge, and the movement of the hyoid bones occurs primarily to facilitate mastication and swallowing.^1^ Temporohyoid osteoarthropathy (THO) is a disease whereby proliferative bone forms around the opposing aspects of the stylohyoid and temporal bones, resulting in gradual decreased joint motion and eventual ankylosis.^2^, ^4^, ^5^, ^6^ The stylohyoid process sheath (SPS) is a part bony, part membranous connective tissue sleeve that surrounds the temporohyoid joint and may provide a scaffold for the mineralisation and enlargement of the joint, which occurs with THO.^7^ On computed tomography (CT), osseous proliferation of increased severity is associated with fracture of the temporal bone^8^ or, less commonly, the stylohyoid bone.^5^ In some cases, there can also be enlargement of the ceratohyoid‐stylohyoid articulation.^9^ The pathogenesis of THO is uncertain; however, increasing severity of osseous proliferation around the THJ has been shown to be associated with increased age^2^, ^7^ and therefore, a degenerative aetiology is likely. Additionally, THO has been proposed to be associated with a transient otitis media and local osteitis^10^, ^11^ or trauma.^6^ The clinical signs associated with THO include headshaking, dysphagia, resenting the bit, crib biting, facial nerve paralysis, vestibular ataxia, seizures, and epistaxis.^12^

Difficulty performing diagnostic anaesthesia of this structure means that diagnostic imaging, often in the form of CT, is used to assess if changes are likely to be clinically significant. However, there is a lack of information to determine what degree of change should be considered significant and what variation can be present in a population of horses with no clinical signs relating to the temporohyoid joint. This study aims to describe the range of computed tomographic features of the equine temporohyoid joint in a referral population undergoing head CT and investigate the association between CT change and possible risk factors. The working hypotheses were that (i) horses will most commonly have low‐grade bone proliferation and cartilage change, (ii) increased grade of CT change will show association with presenting complaints previously reported with THO (e.g., head shaking, neurological deficits).

## 2 MATERIALS AND METHODS

### 2.1 Case selection

In this cross‐sectional study, hospital records for all horses undergoing CT examination of the head and cervical spine (which included the THJs) at the Royal Veterinary College Equine Referral Hospital between November 2017 and December 2021 were reviewed. This included all examinations that were reconstructed with a 0.6 mm slice thickness, as prior to this date thicker slice reconstructions were used. As such, this was a convenience sample. CT examinations with motion artefact or those which did not completely include the temporal and stylohyoid bones were excluded. If horses had multiple CT examinations, the initial CT examination was included, and subsequent examinations were excluded.

### 2.2 Data recording and analysis

CT examinations of the head were acquired either under standing sedation or general anaesthesia, while all examinations of the cervical spine were performed under general anaesthesia. All images were obtained using a 16‐slice multidetector CT scanner, using 120 kV, 400 mA, and a 650 mm field of view (GE Medical Systems, LightSpeed Pro 16). Images were reconstructed using a 512 × 512 matrix, 0.6 mm slice thickness, 0.3 mm slice interval, in a bone window (WW: 2800, WL: 800), with a bone kernel. DICOM images were stored on the hospital picture archiving and communications system and viewed using the same data retrieval system (Horos v3.3.5).

Case records were reviewed and the age, sex, breed, presenting complaint, and primary CT diagnosis of each horse were recorded. The CT diagnosis was recorded from imaging reports written by an ECVDI certified radiologist. Therefore, the CT diagnosis is based on the clinical information and imaging findings at the time of the examination. Presenting complaints and CT diagnoses where similar were grouped into broader categories, as shown in Tables S1 and S2. For example, dental abnormality encompassed presenting complaints such as halitosis, quidding, and dental disease present on an oral examination.

All CT examinations (*n* = 424) were randomised and graded by a large animal radiology resident (R.F.D.) who was blinded to all other variables. To facilitate the training of this large animal radiology resident, a sample population of 20 horses was selected which demonstrated a wide range of severities of bony proliferation and cartilage changes. The CT examinations of these horses were independently reviewed by three authors including the large animal radiology resident (R.F.D.), an ECVDI certified radiologist with 14 years' experience (R.E.M.) and a ECVS certified equine surgeon with 27 years' experience (J.D.P.). Temporohyoid joint characteristics were recorded using the same protocol, with the grading schemes that are described below. Images and results were then discussed, and a consensus was reached on each joint. This process was repeated with 20 further cases. These 40 cases were included in the final study population as they would be difficult to recall in the large dataset.

**FIGURE 1:**
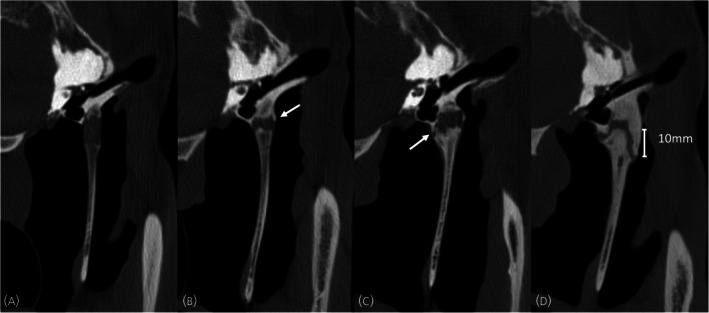
Transverse computed tomographic images at the level of the temporohyoid joint demonstrating the bone grades. (A) Grade 0: no bone proliferation, (B) grade 1: proliferation not bridging the joint (white arrow), (C) grade 2: proliferation bridging the joint (white arrow), (D) grade 3: proliferation extending over 1 cm (digital calliper). Helical acquisition, 120 kV and 400 mA, 512 × 512 matrix, 0.625 mm slice thickness 0.3 mm slice interval, WW: 2800, WL: 800, bone kernel reconstruction.

**FIGURE 2:**
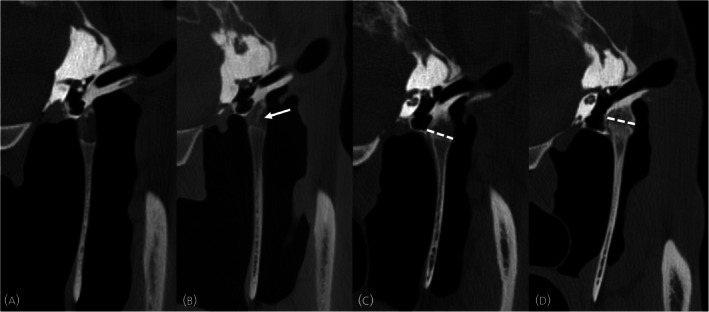
Transverse computed tomographic images at the level of the temporohyoid joint demonstrating the cartilage grades. (A) Grade 0: homogenous attenuation of the cartilage, (B) grade 1: heterogenous attenuation of the cartilage (white arrow), (C) grade 2: 50% ossification of the cartilage, to the level of the tympanic bulla (dotted line), (D) grade 3: complete ossification of the cartilage, to the level of the temporal bone (dotted line). Helical acquisition, 120 kV and 400 mA, 512 × 512 matrix, 0.625 mm slice thickness 0.3 mm slice interval, WW: 2800, WL: 800, bone kernel reconstruction.

**FIGURE 3:**
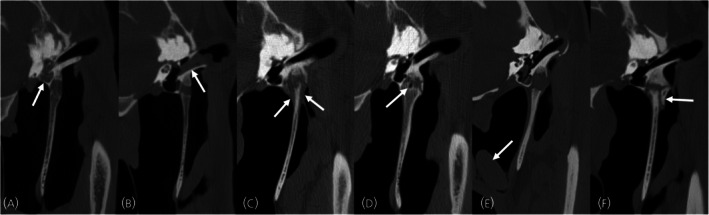
Transverse computed tomographic images at the level of the temporohyoid joint demonstrating the additional imaging findings that were recorded. (A) Otitis media, (B) otitis externa, (C) soft tissue swelling, (D) gas attenuation within the joint, (E) guttural pouch abnormalities (chondroids), (F) fragmentation associated with the joint. Helical acquisition, 120 kV and 400 mA, 512 × 512 matrix, 0.625 mm slice thickness 0.3 mm slice interval, WW: 2800, WL: 800, bone kernel reconstruction.

The characteristics of both temporohyoid joints were recorded separately using a previously reported grading system for the bones^8^ (Figure 1; Table 1) and a novel grading system for the tympanohyoid cartilage (Figure 2; Table 1). This grading scheme was based on previously reported histological changes such as mineralisation within the cartilage.^2^ A sum grade was also created to give a global score for each joint by summing the values of the cartilage and bone grades (e.g., bone grade 2 and cartilage grade 1 results in a sum grade of 3). The location of any osseous proliferation was recorded as present or absent on the temporal bone, stylohyoid bone, or both. Osseous proliferation was recorded as present or absent at the rostral, caudal, lateral, and medial aspects of the joint. Additional imaging features were recorded as present or absent and included evidence of otitis media, otitis externa, local soft tissue swelling, the presence of gas within the joint, guttural pouch abnormalities, and fragmentation or fracture of either the temporal or stylohyoid bones (Figure 3). When recording all the data, structures were assessed in three planes, using multiplanar reconstruction.

**TABLE 1:** Criteria for the bone grade and cartilage grading schemes (see Figures 1 and 2 for example images).

Grade	Bone grade	Cartilage grade
0	No bone proliferation	Homogenous attenuation of the cartilage
1	Bone proliferation not bridging the joint	Heterogenous attenuation of the cartilage
2	Bone proliferation bridging the joint	50% ossification of the cartilage
3	Bone proliferation extending 1 cm or more	100% ossification of the cartilage

CT evidence of otitis media and otitis externa was defined as any soft tissue or fluid attenuating material in the tympanic bulla and external acoustic meatus, respectively.^13^ Soft tissue swelling was considered present if there was subjectively increased soft tissue volume surrounding the portion of the stylohyoid bones within the guttural pouches, the tympanohyoid cartilage and/or the styloid process of the petrous temporal bone. The presence of gas was defined as any hypoattenuating foci within the joint that were isoattenuating to known areas of gas. Guttural pouch abnormalities included findings such as internal fluid, chondroids, and local soft tissue emphysema. A fracture was defined as a hypoattenuating line through the bone that did not correspond to normal canals or sutures,^8^, ^14^ and fragmentation was defined as any mineral attenuating foci associated with the joint that could be viewed as discreet in all three planes of a multiplanar reconstruction.

To gather follow‐up information, a questionnaire was emailed to owners of all horses included in the study (Questionnaire S1). Owners were asked if their horse developed certain clinical signs previously reported to be associated with THO,^4^, ^15^, ^16^ at any time since the head CT. These included facial nerve paralysis, head tilt, ataxia, circling, dysphagia, head shaking, otitis, epistaxis, or a suspicion of head trauma. This questionnaire was sent twice over a two‐month period. If no response was obtained, then the owners were called at random until greater than 200 responses were recorded.

### 2.3 Data analysis

The statistical analysis was performed by a resident with postgraduate training in statistics (R.F.D.) under the guidance of an associate professor in statistics (Y‐M.C.). Descriptive statistics were performed, and outcomes were presented as counts and percentages. An initial univariate analysis was performed, with generalised estimating equations (SPSS Statistics 28.0.0.0 IBM software) used to evaluate risk factors (age, sex, breed, presenting complaint, CT diagnosis, other imaging findings, follow‐up clinical signs) for increased bone, cartilage, and sum grade using the ordinal logistic link function. Due to the low frequency of horses with a sum grade of 5 and 6, these groups were combined to create a 5+ grade for the analysis. An exchangeable correlation matrix was used to account for repeated measures (two joints from the same horse). Odds ratios and 95% confidence intervals were calculated. Type I error rate was set at 5%. Statistically significant risk factors were then incorporated into the multivariate analysis, where the same method was used to perform ordinal logistic regression in a forward stepwise approach, resulting in a multivariable model for each grading system. Any risk factors that were not significant in the multivariable model were considered insignificant, again with type I error rate set at 5%.

## 3 RESULTS

### 3.1 Descriptive

A total of 480 horses met the inclusion criteria. However, 36 cases were excluded due to incomplete inclusion of the THJ and a further 20 cases due to motion artefact, leaving 424 horses (848 THJ) in the study. Sixty‐two percent (263/424) were male and 38% (161/424) female, with a median age of 12 years (range 4 months–29 years, interquartile range [IQR] 8–16 years). There were 44% (186) Warmbloods, 19% (81) Welsh ponies, 12% (50) Thoroughbreds, 8% (36) Cobs, 4% (16) Draught horses, 2% (10) Arabians, 1% (4) cross‐breeds and 10% (41) horses where the breed was not recorded.

The most common presenting complaint was nasal discharge or epistaxis in 21% (90/424) of horses, followed by dental abnormalities in 17% (71/424). Headshaking was reported in 10% (43/424) and cranial nerve deficits were present in 4% (19/424) of horses. The complete frequency distribution of the presenting complaints is presented in Table S1. The most common CT diagnosis was dental disease in 36% (151/424) of horses. Temporohyoid osteoarthropathy was diagnosed in 3% (14/424). The complete frequency distribution of the CT diagnoses is presented in Table S2.

The frequency distribution of the bone, cartilage, and sum grades is presented in Table 2. The most frequently observed (mode) bone grade, cartilage grade and sum grade were 2, 0 and 2, respectively. Complete ankylosis of the stylohyoid joint was not present in any horse. The sum grade was bilaterally symmetrical in 78% (330/424) of horses. Between pairs of THJs, the sum grade differed by one grade in 19% (83/424) and by two grades or more in 3% (11/424) of horses.

**TABLE 2:** Frequency distribution of the different bone grades, cartilage grades and sum grades.

Grade	Bone grade		Cartilage grade		Sum grade	
	Count	Percentage	Count	Percentage	Count	Percentage
0	101	11.9	446	52.6	85	10.0
1	315	37.1	238	28.1	228	26.9
2	355	41.9	148	17.4	229	27.0
3	77	9.1	16	1.9	143	16.9
4					108	12.7
5+					55	6.5

The location of osseous proliferation originated from the temporal bone in 49% (415/848) of joints, the stylohyoid bone in 6% (49/848) and both bones in 33% (281/848). Osseous proliferation was most frequently observed at the medial aspect of the joint in 71% (601/848) of the joints, followed by caudal in 66% (562/848). The most frequently observed additional imaging feature was soft tissue swelling, present in 5% (44/848) of horses, followed by gas in the temporohyoid joint in 3% (25/848). Temporal bone fragmentation was only observed in horses with a sum grade of 4 or greater, and in 1% (6/424) of cases. Stylohyoid bone fracture was present in three horses, while no imaging evidence of a temporal bone fracture was present in any horse. The location of osseous proliferation and the additional imaging features are presented in cross‐tabulation with the sum grade in Tables 3 and 4, respectively.

**TABLE 3:** Cross tabulation of the aspect of the joint at which osseous proliferation is present and the sum grade.

Sum grade	Lateral		Medial		Rostral		Caudal	
	Count	Percentage	Count	Percentage	Count	Percentage	Count	Percentage
0	0	0.0	0	0.0	0	0.0	0	0.0
1	2	0.2	128	15.1	87	10.3	138	16.3
2	11	1.3	181	21.3	88	10.4	161	19.0
3	21	2.5	134	15.8	76	9.0	114	13.4
4	39	4.6	103	12.1	74	8.7	95	11.2
5+	40	4.7	55	6.5	49	5.8	54	6.4
Total	113	13.3	601	70.8	374	44.1	562	66.3

**TABLE 4:** Cross tabulation of the additional imaging features and the sum grade.

Sum grade	Temporal bone fragmentation	Stylohyoid bone fracture	Otitis media	Otitis externa	Gas in the temporohyoid joint	Soft tissue swelling	Guttural pouch changes
0	0	0	0	1	0	0	1
1	0	0	4	2	3	2	0
2	0	1	8	2	3	13	4
3	0	1	4	3	4	7	1
4	1	1	4	2	5	11	3
5+	5	0	3	3	10	11	3
Total	6	3	23	13	25	44	12

Thirty‐four percent (143/424) of owners responded to the questionnaire. Follow‐up information was obtained by telephone conversation for a further 14% (60/424) of the horses, giving a total response rate of 48% (203/424). The most common clinical sign noted by an owner since the head CT was acquired was the suspicion of head trauma in 26% (52/203) of horses, followed by ataxia in 20% (40/203). The complete frequency distribution of the follow‐up clinical signs is presented in Table S3.

### 3.2 Advanced statistical analysis

The results of the univariate analysis are presented in Table S4. The final multivariable model including odds ratios (OR) and *p* values for the bone cartilage and sum grades is presented in Table 5. The variables that remained significant in the multivariate analysis included increased age, which was positively associated with an increase in all grading schemes (*p* < 0.001). The Thoroughbred breed was positively associated with an increase in all grading schemes (*p* bone 0.02, cartilage <0.001, sum <0.001), and the Arabian was positively associated with an increased bone (*p* < 0.001) and sum grade (*p* 0.02). The Cob breed was negatively associated with an increase in cartilage (*p* < 0.03) and sum grade (*p* < 0.001). Proliferation from both bones, temporal bone fragmentation, and soft tissue swelling were all positively associated with increased bone grade (*p* < 0.001, <0.001, 0.009, respectively). A diagnosis of THO was positively associated with increased cartilage and sum grade (*p* < 0.001 for both). There was no presenting complaint or follow‐up clinical sign that was significantly associated with an increase in any grading scheme in the multivariable analysis.

**TABLE 5:** Multivariable model for the bone, cartilage and sum grades.

Risk factor	Bone grade			Cartilage grade			Sum grade		
	Odds ratio	95% CI	*p* Value	Odds ratio	95% CI	*p* Value	Odds ratio	95% CI	*p* Value
Age (per year)	1.08	1.04–1.12	<0.001	1.09	1.05–1.13	<0.001	1.09	1.05–1.12	<0.001
Sex									
Male	Ref								
Female	1.62	1.09–2.42	0.018						
Breed									
Warmblood	Ref			Ref			Ref		
Thoroughbred	1.94	1.09–3.47	0.025	3.28	1.71–6.31	<0.001	2.87	1.53–1.12	<0.001
Arab	6.69	2.27–19.73	<0.001				4.20	1.26–13.95	0.02
Cob				0.44	0.21–0.93	0.03	0.29	0.15–0.57	<0.001
Proliferation: Both									
No	Ref								
Yes	2.47	1.56–3.92	<0.001						
Temporal bone frag.									
No	Ref								
Yes	26.83	5.09–141.44	<0.001						
Soft tissue swelling									
No	Ref								
Yes	1.92	1.18–3.13	0.009						
Diagnosis of THO									
No				Ref			Ref		
Yes				14.07	3.11–63.61	<0.001	9.23	2.54–33.47	<0.001

Bony proliferation was least frequently observed on the lateral aspect of the joint, and in the univariate analysis, this lateral proliferation was positively associated with increased sum grade (OR 15.8, 95% CI 9.1–27.5, *p* < 0.001). This did not remain significant in the multivariable model as lateral proliferation was significantly associated with age and breed (*p* < 0.001). The only follow‐up clinical sign that was significant in the univariate analysis was ataxia, which was positively associated with the sum grade (OR 2.459, 95% CI 1.0–5.8, *p* 0.04), this did not remain significant in the multivariable model. In the cross tabulation (Table 4), gas in the temporohyoid joint occurred more frequently in the higher sum grades; however, this was not significantly associated with an increase of any grade in the univariate analysis.

## 4 DISCUSSION

The findings of this study suggest that moderate osseous proliferation at the temporohyoid joint is a common finding in horses presenting for unrelated disease, with bone grade 2 being the most frequently observed. However, tympanohyoid cartilage ossification was uncommon in this group of horses, with cartilage grade 0 being most frequently observed. Osseous proliferation most commonly originated from the temporal bone and was more often bilaterally symmetrical. Osseous proliferation was most common on the medial and caudal aspects of the joint. Lateral proliferation was rare and positively associated with increased sum grade in the univariate analysis, and so may be of clinical importance. Increased age was positively associated with an increase in all grading schemes, but the presence of otitis media or externa was not. For the first time, an association between increased grade and certain breeds was observed in this study, which suggests that Thoroughbreds and Arabians may be predisposed to greater THJ remodelling, while the Cob breed may be protective. There was no association between any presenting complaint and an increase in any grading scheme; therefore, it was not possible to prove an association between greater THJ remodelling and headshaking. Some imaging findings were associated with increased bone grade, such as soft tissue swelling and temporal bone fragmentation. Gas in the THJ increased with increasing sum grade, and although this finding was not statistically significant, it may be clinically important. Finally, the only CT diagnosis which was associated with increased cartilage or sum grade was a diagnosis of THO, which suggests the reporting diagnostic imaging specialist and the observer in this study tended to agree. Additionally, increased bone grade was not associated with a diagnosis of THO, but increased cartilage was, which suggests that cartilage change may occur with more severe disease. The lack of association with the bone grade is likely because bony change was common in all horses. The results of this study only partially support the hypothesis (i) horses will most commonly have low‐grade bone proliferation and cartilage change; horses commonly had moderate‐grade bone proliferation, but change to the tympanohyoid cartilage was uncommon. The second hypothesis (ii) increased grade of CT change will show an association with presenting complaints previously reported with THO was rejected. There was no association between increased grade and presenting complaints previously reported to be associated with THO.

In the follow‐up questionnaire, the only clinical sign which was significantly associated with increased sum grade was ataxia, but this did not remain significant in the multivariable model. It should also be recognised that this data was acquired from owners and therefore it may be less reliable. However, it is well documented that horses with more severe THO are more likely to develop a temporal bone fracture resulting in vestibular ataxia.^8^ Therefore, it remains possible that horses with more bone and cartilage change at the THJ may be more likely to develop clinical THO and subsequent ataxia, and so the findings of this study are in keeping with this known association. Due to the method of data collection, the aetiology of ataxia in these cases is unknown and may be the result of THO or cervical issues amongst other causes. Further work is needed to establish what degree of remodelling of the THJ is likely to result in future clinical signs.

The pathogenesis of THO remains the subject of discussion. In the recent literature, it has been demonstrated that there is a positive association between increasing age and increased severity of THO.^2^, ^7^ The results of the present study provide further evidence to support this, and therefore the theory that THO is a degenerative condition. It has also been proposed that THO may be a sequala of a transient otitis media and interna. This is based on an abstract describing three horses with THO and fractures of the petrous temporal bone that underwent a necropsy and were found to have proteinaceous fluid in the tympanic bulla and evidence of bacterial meningitis.^11^ The present study found no association between imaging evidence of otitis media or externa and THO. Although it may be possible for a transient otitis to cause THO, these results would suggest that this is not common. Otitis interna was not assessed in this study as it is more accurately assessed with magnetic resonance imaging and therefore this is a potential indication for this modality.^17^ Several cases in the present study demonstrated evidence of gas within the THJ. This may be the result of infection but also may be caused by vacuum phenomenon or trauma.^10^, ^18^, ^19^

The SPS is a connective tissue sleeve surrounding the temporohyoid joint and may provide a scaffold for the mineralisation which occurs with THO.^7^ In a recent study, it was found that the ossified portion of the SPS was larger medially than laterally in a population of horses presenting for head CT examination. The results of the present study support this finding, and in addition show that caudal osseous proliferation is also frequently observed. Mild caudomedial proliferation is therefore likely to be normal, while lateral proliferation is rare and associated with increased sum grade, suggesting this is likely to be more abnormal.

In a study examining the association between THO and temporal bone fracture, it was found that 41% (16/39) horses with THO had complete fusion of the temporohyoid joint.^8^ In the present study population, no horses were found to have fusion of the THJ. This is likely a result of the differing inclusion criteria, as in the previous study only horses with clinical signs of THO were included. However, the present study would suggest that complete fusion is uncommon.

In the present study, a sample size calculation was not performed due to the predetermined number of CT examinations that were available for retrospective review. Other limitations of this study include the use of a single observer and the lack of histological confirmation of the findings. However, it is believed that appropriate measures were taken to ensure the observer was able to grade the images consistently, and there is published data which demonstrates good agreement between CT findings of THO and histology.^2^ The inclusion of the training cases in the final dataset is another limitation, as it is possible that the observer could have recalled the cases; however, this was considered unlikely. Finally, the outcomes regarding breed will be population‐dependent. In this study, the breed distribution was varied, but there were low numbers of certain breeds, such as Arabians, and therefore this may affect the reliability of the results.

In conclusion, moderate osseous proliferation is common in horses presenting with unrelated clinical signs; therefore, mild to moderate bony change is unlikely to be clinically significant. However, cartilage ossification was uncommon and was associated with a CT diagnosis of THO, so particular attention can be paid to the cartilage as an indicator of an abnormal joint. Similarly, lateral osseous proliferation, soft tissue swelling, temporal bone fragmentation, and gas in the THJ may serve as markers of disease. Thoroughbreds and Arabian horses are at increased risk of developing THO. Increased grade was associated with increased age but not with the concurrent imaging finding of otitis, suggesting THO is predominantly a degenerative disease.

## FUNDING INFORMATION

Clients who responded to the questionnaire were entered into a draw for a £50 Amazon voucher. Funding for this study was provided by the authors.

## CONFLICT OF INTEREST STATEMENT

The authors declare no conflicts of interest.

## AUTHOR CONTRIBUTIONS

**Rupert F. Dash:** Conceptualization; methodology; data curation; investigation; formal analysis; visualization; writing – original draft; writing – review and editing. **Justin D. Perkins:** Conceptualization; methodology; data curation; investigation; formal analysis; supervision; writing – original draft; writing – review and editing. **Yu‐Mei Chang:** Conceptualization; methodology; data curation; supervision; formal analysis; investigation; writing – original draft; writing – review and editing; project administration. **Rhiannon E. Morgan:** Conceptualization; methodology; data curation; supervision; formal analysis; investigation; project administration; writing – review and editing; writing – original draft.

## DATA INTEGRITY STATEMENT

R. F. Dash and Y.‐M. Chang had full access to all the data in the study and take responsibility for the integrity of the data and the accuracy of the data analysis.

## ETHICAL ANIMAL RESEARCH

Approved by the Clinical Research Ethical Review Board of the Royal Veterinary College.

## INFORMED CONSENT

Owner consent was obtained for the use of images and data from the medical record in research in general. Horse owners who completed a follow‐up questionnaire gave consent for the use of these data in this study.

## Supporting information


**Data S1:** Questionnaire S1: RVC Equine Research Questionnaire.

**Table S1:** Frequency distribution of the presenting complaints of all horses (*n* = 424).

**Table S2:** Frequency distribution of the CT diagnosis of all horses (*n* = 424).

**Table S3:** Frequency distribution of the follow‐up clinical signs (horses *n* = 203).

**Table S4:** Univariate analysis, with significant risk factors presented in bold text.

## Data Availability

The data that support the findings of this study are openly available in Figshare at http://doi.org/10.6084/m9.figshare.28542683.
