# Supplementary material for: Computed tomography of the equine temporohyoid joint: Association between imaging changes and potential risk factors
Source: Equine Vet J. 2025 May 5;58(1):125–33. doi: 10.1111/evj.14495 (PMC12699099; doi:10.1111/evj.14495)
Supplement: Supplementary file 1 — Data S1: Questionnaire S1: RVC Equine Research Questionnaire. [file EVJ-58-125-s003.pdf]

Equine Veterinary Journal BEVA

Questionnaire S1: Questionnaire.

RVC Equine Research Questionnaire

PLEASE READ THE FOLLOWING CAREFULLY BEFORE COMPLETING THE QUESTIONNAIRE.

Thank you very much for taking part in this questionnaire. Your answers will provide valuable information to our team and help improve horse welfare in the future.

In the third question we must unfortunately ask if your horse has been put down for any reason since their visit(s) to the RVC. This is important to know, and you can still participate in the rest of the questionnaire if your horse has unfortunately been put to sleep.

In the sections that follow, please tick 'yes' or 'no' to indicate if your horse has ever shown any of the symptoms listed in that section at any time since the visit to the RVC. The symptoms in each section are related, but even if your horse has only shown one of the symptoms listed, please tick the 'yes' box.

What is your full name?

What is your horses name?

If your horse has been put down since visiting the RVC please could you tell us why? If this does not apply to you please move on to section 1.

Section 1: Facial nerve paralysis, drooping of one side of the face, inability to move one side of the face including the eyelid or ear.

Section 2: A head tilt, holding the head slightly asymmetrically or rotated with the pol or mouth consistently held to one side.

Section 3: Ataxia, wobbly or uncoordinated gate, tripping frequently, exaggerated limb movements, almost falling or falling down.

Section 4: Difficulty swallowing (excluding if caused by known dental disease).

Section 5: Possible unseen head trauma, any suspicion that the horse has injured its head, behaving as if pain is originating from the head.

Section 6: Head shaking, shaking or tossing the head around, when being worked or at rest.

Section 7: Ear infection, being diagnosed with an infection of the ear by your vet, discharge from the ear.

Section 8: Haemorrhage (bleeding) from the ear.

Section 9: Circling; if the horse repeatedly walks in circles and cannot be stopped. This would likely result in you calling your vet quickly.

Section 10: Bleeding from both nostrils

Question 11: If your horse has been euthanised for any of the above reasons which of these signs did they show?

Question 12: If your horse has been retired for any of the above reasons which of these signs did they show?
