# Supplementary material for: Computed tomography of the equine temporohyoid joint: Association between imaging changes and potential risk factors
Source: Equine Vet J. 2025 May 5;58(1):125–33. doi: 10.1111/evj.14495 (PMC12699099; doi:10.1111/evj.14495)
Supplement: Supplementary file 2 — Table S1: Frequency distribution of the presenting complaints of all horses (n = 424). [file EVJ-58-125-s004.pdf]

Equine Veterinary Journal BEVA

Table S1: Frequency distribution of the presenting complaints of all horses (n = 424).

Presenting Complaint	Number of horses	
	Count	Percentage
Nasal discharge/epistaxis	90	21.2%
Dental Abnormality	71	16.7%
Mass/swelling	57	13.4%
Head shaking	43	10.1%
Dysphagia/decreased appetite	38	9.0%
Neurological	38	9.0%
Gait abnormality/poor performance	29	6.8%
Pain other	20	4.7%
Cranial nerve deficits	19	4.5%
Trauma	10	2.4%
Ophthalmic abnormality	5	1.2%
Aural abnormality	4	0.9%
