# Supplementary material for: Computed tomography of the equine temporohyoid joint: Association between imaging changes and potential risk factors
Source: Equine Vet J. 2025 May 5;58(1):125–33. doi: 10.1111/evj.14495 (PMC12699099; doi:10.1111/evj.14495)
Supplement: Supplementary file 3 — Table S2: Frequency distribution of the CT diagnosis of all horses (n = 424). [file EVJ-58-125-s005.pdf]

Equine Veterinary Journal BEVA

Table S2: Frequency distribution of the CT diagnosis of all horses (n = 424).

CT diagnosis	Number of horses	
	Count	Percentage
Dental disease	151	35.6%
No diagnosis	75	17.7%
Abscess/haematoma/mass	46	10.8%
Trauma/fracture	33	7.8%
Sinusitis	27	6.4%
Cervical osteoarthritis	23	5.4%
Neurological disease	15	3.5%
Temporohyoid osteoarthropathy	14	3.3%
Otitis (media and/or externa)	11	2.6%
Suture periostitis	9	2.1%
Cervical disease	6	1.4%
Temporomandibular joint disease	5	1.2%
Laryngeal/guttural pouch disease	4	0.9%
Sialoadenitis	3	0.7%
Ophthalmic disease	2	0.5%
