# Supplementary material for: Computed tomography of the equine temporohyoid joint: Association between imaging changes and potential risk factors
Source: Equine Vet J. 2025 May 5;58(1):125–33. doi: 10.1111/evj.14495 (PMC12699099; doi:10.1111/evj.14495)
Supplement: Supplementary file 4 — Table S3: Frequency distribution of the follow‐up clinical signs (horses n = 203). [file EVJ-58-125-s001.pdf]

Equine Veterinary Journal BEVA

Table S3: Frequency distribution of the follow-up clinical signs (horses n = 203).

Follow up clinical sign	Number of horses	
	Count	Percentage
Suspicion of head trauma	52	26%
Ataxia	40	20%
Head tilt	38	19%
Dysphagia	22	11%
Otitis	14	7%
Epistaxis	14	7%
Circling	12	6%
Facial nerve paralysis	4	2%
