# Supplementary material for: Computed tomography of the equine temporohyoid joint: Association between imaging changes and potential risk factors
Source: Equine Vet J. 2025 May 5;58(1):125–33. doi: 10.1111/evj.14495 (PMC12699099; doi:10.1111/evj.14495)
Supplement: Supplementary file 5 — Table S4: Univariate analysis, with significant risk factors presented in bold text. [file EVJ-58-125-s002.pdf]

Equine Veterinary Journal BEVA

Table S4: Univariate analysis, with significant risk factors presented in bold text.

		**Bone Grade**				**Cartilage Grade**				**Bone/Cartilage sum Grade**		
		**95% Confidence interval**				**95% Confidence interval**				**95% Confidence interval**		
**Risk factors**	**OR**	**Lower**	**Upper**	**p Value**	**OR**	**Lower**	**Upper**	**p Value**	**OR**	**Lower**	**Upper**	**p Value**
Age	**1.064**	**1.032**	**1.098**	**<0.001**	**1.073**	**1.040**	**1.106**	**<0.001**	**1.070**	**1.039**	**1.101**	**<0.001**
**Sex**												
Male	1.000	.	.	.	1.000	.	.	.				
Female	1.391	0.980	1.975	0.065	1.355	0.943	1.949	0.101	**1.468**	**1.043**	**2.069**	**0.028**
**Breed**	**Across all**			**<0.001**	**Across all**			**0.004**	**Across all**			**<0.001**
Warmblood	1.000	.	.	.	1.000	.	.	.	1.000	.	.	.
Thoroughbred	1.658	0.953	2.883	0.073	**3.055**	**1.718**	**5.432**	**<0.001**	**2.515**	**1.469**	**4.303**	**<0.001**
Pony	0.853	0.534	1.362	0.505	0.866	0.525	1.432	0.574	0.908	0.573	1.440	0.683
Draught horse	0.563	0.241	1.362	0.184	1.116	0.455	2.742	0.810	0.774	0.312	1.919	0.581
Cross Breed	1.803	0.911	3.566	0.910	0.682	0.133	3.483	0.645	1.281	0.546	3.004	0.570
Cob	**0.471**	**0.228**	**0.926**	**0.041**	0.759	0.356	1.621	0.477	**0.486**	**0.246**	**0.958**	**0.037**
Arab	**5.971**	**1.984**	**17.969**	**0.001**	1.862	0.464	7.463	0.380	**3.686**	**1.215**	**11.187**	**0.021**
